# HDAC4 degradation by combined TRAIL and valproic acid treatment induces apoptotic cell death of TRAIL-resistant head and neck cancer cells

**DOI:** 10.1038/s41598-018-31039-8

**Published:** 2018-08-21

**Authors:** Bok-Soon Lee, Yeon Soo Kim, Haeng-Jun Kim, Dae-Ho Kim, Ho-Ryun Won, Yong-Sung Kim, Chul-Ho Kim

**Affiliations:** 1 Department of Otolaryngology, School of Medicine, Ajou University, Suwon, 16499 Republic of Korea; 2 Department of Otorhinolaryngology, College of Medicine, Konyang University Hospital, Konyang University Myunggok Medical Research Institute, Daejeon, 35365 Republic of Korea; 3 Department of Molecular Science and Technology, Ajou University, Suwon, 16499 Republic of Korea; 4 Department of Otolaryngology-Head and Neck Surgery, Research Institute for Medical Science, Chungnam National University, Daejeon, 35015 Republic of Korea

## Abstract

Although TRAIL can directly induce cell death in some cancer cells, it appears that TRAIL resistance exists in many cancers. This study focuses on anti-cancer drugs for TRAIL-resistant head and neck cancer (HNC) to provide further progress toward effective cancer therapy. Results indicate in TRAIL-resistant HNC cells, that combined TRAIL and VPA treatment greatly reduced cell viability and therefore induced cell death, relative to treatment with TRAIL or VPA alone. A caspase-dependent signaling pathway was demonstrated, and combined treatment with TRAIL and VPA also significantly decreased the expression of HDAC4. When we pretreated cells with z-VAD followed by combined treatment with TRAIL and VPA, cell death was blocked with no reduction in expression of HDAC4. To confirm that cell death involved HDAC4 in HNC cells, we knocked down expression of HDAC4 with siRNA, followed by treatment with TRAIL and VPA. Results showed that loss of HDAC4 sensitized the TRAIL-resistant HNC cells to apoptotic cell death. Finally, we showed elevated expression of HDAC4 in HNC tissues compared to normal tissues obtained from the same patients. In conclusion, we suggest that combined VPA and TRAIL treatment may be a promising therapy for HNC via HDAC4 degradation.

## Introduction

Head and neck cancer (HNC), including the oral cavity, pharynx, and larynx, is the sixth most common malignant tumor worldwide^1,2^. Such cancers are mainly caused by consuming alcohol and smoking tobacco, resulting in genomic mutations^3^. HNC therapy normally includes surgery, radiation and chemotherapy, however, overall survival rates in recent times have not improved^4^. Therefore, it is essential to develop alternative effective treatment approaches for patients with HNC.

TRAIL (tumor necrosis-factor (TNF)-related apoptosis-inducing ligand) is a member of the TNF family, and it usually induces apoptotic cell death in several different kinds of cancer cells without having an appreciable effect in normal cells^5^. The TRAIL receptors TRAIL-R1 and TRAIL-R2 have also been known as DR4 (death receptor 4) and DR5, respectively^6^. Once TRAIL binds to its receptor, oligomerization recruits downstream molecules, such as FAS-associated death domain (FADD), and is then able to form the procaspase-8 activating death-inducing signaling complex (DISC)^7^. Thus, it has been implicated in triggering cell death physiological responses^7^. However, several studies have reported that many cancers are TRAIL-resistant^8–10^, meaning that TRAIL fails to induce apoptotic cell death^11,12^. More recently, TRAIL has been used in combination therapy with other cancer drugs to overcome this resistance^10^. Of various strategies, some studies have attempted to increase the expression of DR4 or DR5 receptors, improving death-related signals, and preventing survival signaling^13–15^. Others have developed agonistic antibody engineered for DR4 or DR5^16,17^.

HDACs are histone deacetylases that are counteracted by histone/lysine acetyltransferases^18^. There are class I HDACs (HDAC1, 2, 3, 8) and class II HDACs (HDAC4, 5, 6, 7, 9, 10)^19^. Class II HDACs are able to shuttle in and out of the nucleus in response to certain cellular signals^19^. Also, 14-3-3 protein is able to associate with HDAC4/5 or HDAC7 in the cytoplasm when phosphorylated, and these then enter the nucleus^18^. HDAC inhibitors have been known to induce cell cycle arrest, differentiation and apoptosis *in vitro* and *in vivo*^20^. Among HDAC inhibitors, valproic acid (VPA) has been reported to have anti-cancer activity due to transcriptional regulation of cell survival factors^21^.

In this study, we examined VPA to determine the sensitivity of HNC cells to TRAIL, which could result in apoptotic cell death, and in addition we identified the precise cell death mechanism. Our findings provide insights into the potential for combined treatment with TRAIL and VPA, which together induce apoptotic cell death via degradation of HDAC4. Our data suggest this approach has great potential as an anti-cancer strategy for head and neck cancers.

## Results

### VPA induced cell death in TRAIL-resistant head and neck cancer cells

Previously, Sung *et al*. reported several HDAC inhibitors induced the apoptotic cell death in TRAIL-resistant cells^22^. This sounded interesting and a good reference to us. Therefore, we wanted to apply it to this study and examine the head and neck cancer cells lines that are resistant to TRAIL. We selected VPA among HDAC inhibitors to investigate the combined effect of VPA and TRAIL on HNC cells (FaDu, HN3 and SNU899) and performed an MTT assay with cells following treatment with VPA (2.5, 5, 10, 20 mM) and/or TRAIL (0.5, 1 µg/ml) for 24 h.

**Figure 1:**
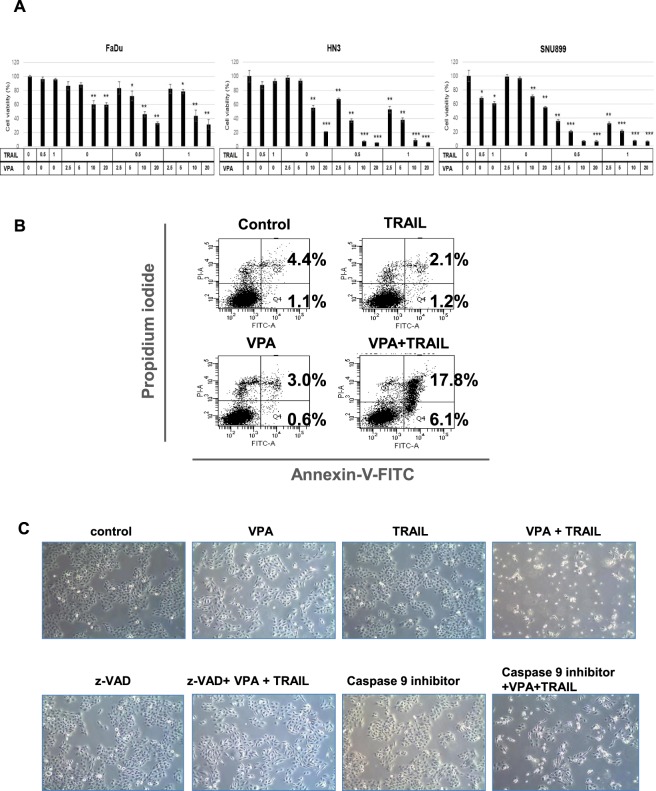
Combined TRAIL and VPA treatment induced cytotoxicity in head and neck cancer cell lines. **(A)** FaDu, HN3 and SNU899 were treated with TRAIL (0.5, 1 µg/ml) and/or VPA (2.5, 5, 10, 20 mM) for 24 h. MTT assay was used to measure cell viabilities. (*p < 0.05; **p < 0.01; ***p < 0.001) **(B)** HN3 cells were treated with TRAIL (0.5 µg/ml) and VPA (5 mM), and then the cells were stained with annexin V-FITC and propidium iodide and analyzed via flow cytometry. **(C)** Cell death by combination TRAIL and VPA was determined using a light microscope.

As shown in Fig. 1A, FaDu and HN3 cells were resistant to TRAIL alone, but cell viability was decreased by combined treatment with VPA and TRAIL, however this was not the case for SNU899 cells. Moreover, cell death from combined VPA and TRAIL treatment was confirmed using Annexin-V/PI staining assay (Fig. 1B).

To examine the involvement of caspase activation in apoptotic cell death, we also pretreated cells with z-VAD (caspase 3, 6, 7 inhibitor) or caspase 9 inhibitor for 1 h followed by treatment with VPA and/or TRAIL for 24 h. We observed apoptotic cells by light microscopy (Fig. 1C) and confirmed that apoptotic cell death by combined treatment of VPA and TRAIL in HNC cells occurred in a caspase-dependent manner.

### Cell surface receptor expression of DR4 and DR5 on HN3 cells

**Figure 2:**
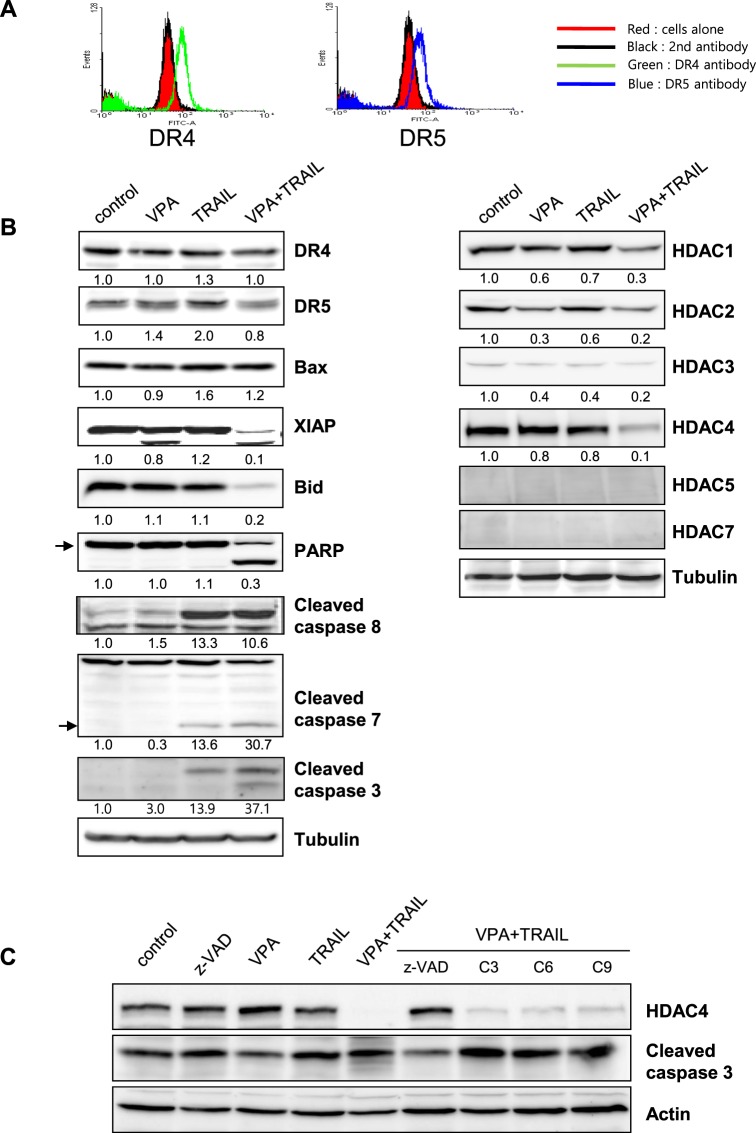
Combined TRAIL and VPA treatment induced caspase -dependent cell death signaling and decreased the expression of HDAC4. **(A)** HN3 cells were harvested, and FACS analysis was performed using anti-DR4 (left) or DR5 (right) antibody. Histograms display the fluorescence intensity (*x*-axis) versus relative cell numbers (*y*-axis). Black line, goat IgG isotype-control-labeled cells; red fill, cells only; green line, DR4-labeled cells; blue line, DR5-labeled cells. **(B)** HN3 cells were treated with TRAIL (0.5 µg/ml) and VPA (5 mM) for 24 h, and then Western blotting was performed with the following antibodies: DR4, DR5, Bax, XIAP, Bid, cleaved PARP, cleaved caspase 8, 7, 3, HDAC1, 2, 3, 4, 5, 7 and tubulin. **(C)** HN3 cells were pretreated with z-VAD for 1 h, followed by combined treatment TRAIL (0.5 µg/ml) and VPA (5 mM) for 24 h. Western blotting was performed with the following antibodies: HDAC4 and cleaved caspase 3.

We tested the expression of functional death receptors (DR4 and DR5) to better understand how the mechanism of cell death through the combined treatment of TRAIL and VPA in HNC cell lines. According to the previous studies, it has been suggested that the upregulation of DR4 and DR5 by cancer drugs increased the TRAIL-induced cell death of cancer cells^23,24^. We assessed cell surface receptor expression of DR4 and DR5 using anti-DR4 and anti-DR5 antibodies on HN3 cells. Although it appeared that there was cell surface expression of DR4 and DR5 on HN3 cells (Fig. 2A), TRAIL and VPA treatment did not increase their expression.

### Combined TRAIL and VPA treatment induced the activation of apoptotic protein

Western blotting was also performed using antibodies against DR4, DR5, Bax, XIAP, Bid, cleaved PARP, cleaved caspase 8, 7 and 3, HDAC 1, 2, 3, 4, 5 and 7 to see the activation of apoptotic molecules in HN3 cells treated with TRAIL and/or VPA. The result showed that proapoptotic molecule BAX slightly increased and antiapoptotic molecules, XIAP and Bid decreased, and apoptotic cell death marker, cleaved caspase 3, cleaved PARP increased during treatment with the TRAIL and VPA combination, consistent with phenotype of the cell death (Fig. 2B). In this experiment, cleaved caspase 8 and cleaved caspase 7 do not seem to be related to the cell death induced by TRAIL/VPA combination, because those already showed the cleaved forms in cells treated with TRAIL alone, without phenotype of cell death. It was interesting enough to bring us a question that it is the combined treatment of TRAIL and VPA that induced the cell death, not TRAIL alone. Therefore we tried to identify the specific mechanism among HDAC molecules and observed the diminished expression of HDAC4 in the cells treated TRAIL and VPA not TRAIL alone. To support this finding, we also pretreated the cells with the related caspase inhibitors (z-VAD, caspase 3, 6 and 9 inhibitors), followed by combined treatment with TRAIL and VPA to find the relation between HDAC4 expression and cell death in HN3 cells (Fig. 2C). The results showed that HDAC4 was completely degraded by the combined TRAIL/VPA treatment and partially degraded with each caspase inhibitor.

### Cell death induced by treatment with TRAIL/VPA affects expression of 14-3-3 proteins

**Figure 3:**
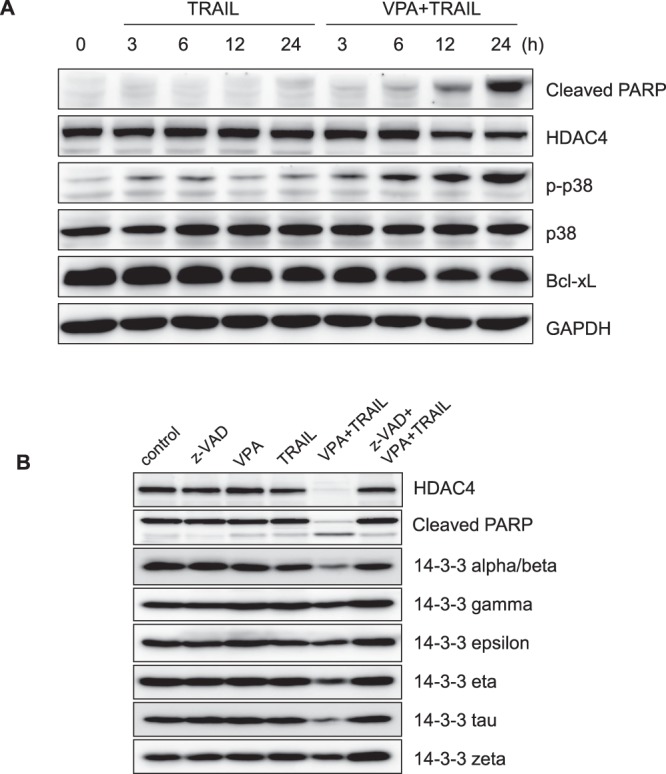
The expression of 14-3-3 proteins were decreased by combined treatment of TRAIL and VPA. **(A)** HN3 cells were treated with TRAIL (0.5 µg/ml) and/or VPA (5 mM) for 3, 6, 12 and 24 h. The lysates were immunoblotted with the following antibodies: cleaved PARP, HDAC4, p-p38, p38 and Bcl-xL. **(B)** HN3 cells were pretreated with z-VAD for 1 h, followed by TRAIL (0.5 µg/ml) and VPA (5 mM) for 24 h. Western blotting was then performed with the following antibodies: HDAC4, cleaved PARP, 14-3-3 alpha/beta, gamma, epsilon, eta, tau and zeta.

We also investigated another related signaling pathway in cell death by the combined treatment with TRAIL and VPA by doing Western blotting after the combined treatment for 3, 6, 12 and 24 h in HN3 cells. The critical roles of p38 or JNK signaling for activating the programmed cell death have been already shown previously^25^. In this experiment, we were able to observe the up-regulation of p-p38 at 6, 12, and 24 h during the cell death unlike the treatment with TRAIL alone. However, JNK signaling did not detected (data not shown), and the expression of Bcl-xL which blocks apoptotic cell death^26^ was decreased. (Fig. 3A).

Previously, Wang *et al*. reported that HDAC4 was regulated by binding to 14-3-3 proteins^27^. To determine the involvement of 14-3-3 proteins in the degradation of HDAC4, we performed Western blotting after combined TRAIL and VPA treatment in HN3 cells. As shown in Fig. 3B, the expression of 14-3-3 proteins decreased during the process of TRAIL/VPA induced cell death (Fig. 3B). However, the binding of HDAC4 and 14-3-3 protein does not appear to be related to apoptosis (data not shown).

### Knock-down of HDAC4 sensitized head and neck cancer cells to TRAIL-induced cell death

**Figure 4:**
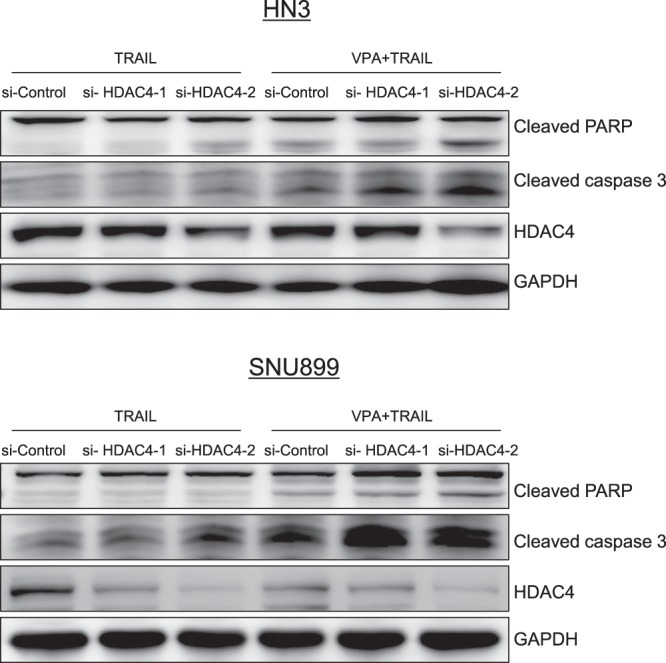
Knock-down of HDAC4 enhanced TRAIL-induced cell death. HN3 and SNU899 cell lines were transfected with HDAC4 siRNA using RNAiMAX transfection reagent. After transfection for 24 h, cells were treated with TRAIL alone or TRAIL (0.5 µg/ml) and VPA (5 mM) for 18 h. Western blotting was performed with anti-cleaved PARP, cleaved caspase 3, HDAC4 and GAPDH antibodies.

It is obvious that highly expressed HDAC4 plays a critical role in TRAIL resistant cells. To confirm and support this fact, we knocked down the HDAC4 expression with HDAC4 siRNA in cells (Fig. 4). After transfection for 24 h, the transfected cells were treated with TRAIL alone or combined treatment of TRAIL and VPA. As a result, the cell death was observed in the TRAIL alone induced cells treated with HDAC4 siRNA, compared to control cells treated with siRNA-treated, which led us to a conclusion that HDAC degradation is important for cell death induced by TRAIL in TRAIL-resistant cells.

### Expression of HDAC4 is upregulated in human HNC tissue

**Figure 5:**
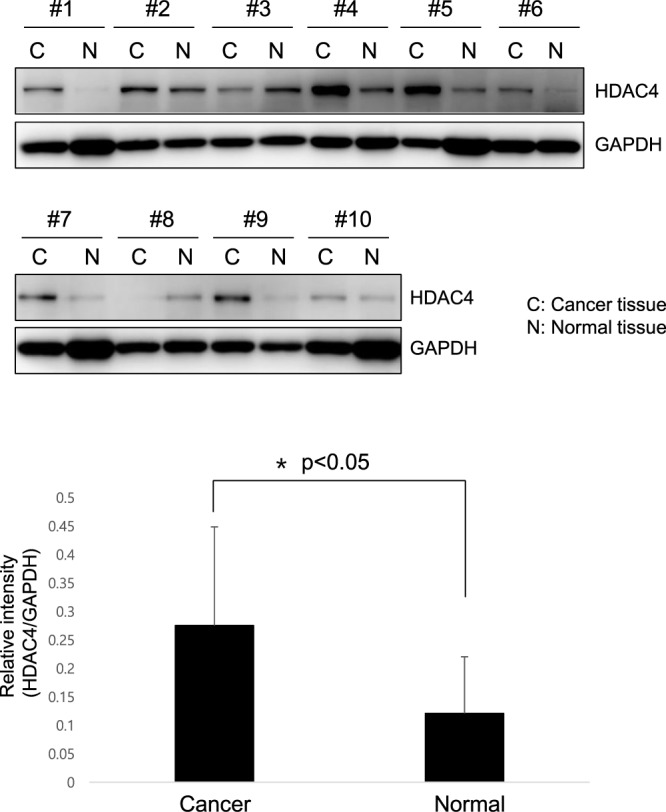
Expression of HDAC4 is upregulated in human HNC tissues and paired normal tissues. Western blotting was performed to examine the protein expression of HDAC4 in head and neck cancer tissues **(C)** compared to normal adjacent tissues (N). (patients n = 10).

We performed more experiments with 10 cancer tissues from head and neck cancer patients. HDAC4 expression was tested by Western blotting with 10 cancer tissues and paired normal tissues. We observed more clear upregulation in HNC tissues compared to normal tissues (Fig. 5). Therefore, HDAC4 could be a good potential therapeutic target for HNC therapy.

## Discussion

Head and neck cancers are highly heterogeneous and very difficult to cure. Therefore, there is an urgent need to develop additional therapeutic agents. TRAIL is a useful ligand to induce death in many cancer cells that overexpress the death receptors (DR4 and DR5) because it does not affect normal cells. Some cancers are resistant however. As a consequence there are many ongoing studies to try to overcome this resistance and discover pathways that achieve the therapeutic goals. Some studies have found an increase in the expression of DR4 or DR5 induced by combined treatment of multiple drugs and reported that TRAIL enhances the cell death of cancer cells. Singh *et al*. showed that chemotherapeutic drugs (paclitaxel, vincristine, vinblastine, etoposide, adriamycin, and camptothecin) induce the expression of DR4 and/or DR5, and combining treatment with TRAIL has been shown to cause apoptotic cell death in an additive or synergistic manner *in vitro*^28^. Also, use of HDAC inhibitors with TRAIL, enhances cell death. According to a study from Schuler *et al*., depletion of HDAC2 sensitizes pancreatic ductal adenocarcinoma to TRAIL-induced apoptosis via upregulation of DR5^14^. Fandy *et al*. also found that HDAC inhibitors, SAHA or TSA enhanced the cytotoxic and apoptotic effects of TRAIL and upregulated the surface expression of DR4 and/or DR5^13^.

Furthermore, Zheng *et al*. have reported that the depletion of c-FLIP by siRNA increased DR5 expression, enhancing T-lymphoma cell apoptosis and sensitizing cell death to chemotherapeutic agents^29^.

TRAIL-mediated apoptosis signaling is definitely a very crucial pathway involved with caspase 8 activity. In this case, as the Fig. 2B shows, the activity of cleaved caspase 8 is shown enough to see the involvement with only TRAIL itself. However, even though the combined treatment of TRAIL and VPA does show the activity of caspase 8, cell death doesn’t occur. Therefore, we suspect that there should be something that controls the activity of effector caspases, which needs further study.

Interestingly, in this study, upregulation of DR4 and DR5 was not seen in the presence of TRAIL and VPA (Fig. 2B), which convinced us that another mechanism was likely involved in TRAIL/VPA induced cell death. Therefore, when we immunoblotted with anti-HDAC 1, 2, 3, 4, 5 and 7 antibodies, we focused more on a decrease in HDAC4 expression in the presence of both TRAIL and VPA, and not TRAIL or VPA alone. We also found that not only did z-VAD inhibit cell death induced by TRAIL/VPA, but also that it stopped HDAC4 degradation.

Consistent with our data, Zeng *et al*. showed that HDAC4 overexpression is important for the oncogenesis of esophageal carcinoma^30^, which supports the belief that the expression of HDAC4 in cancer cells is crucial to the carcinogenic potential.

Nishino *et al*. showed that 14-3-3 proteins regulate the nuclear import of class II a histone deacetylases^31^. To find out that the importance of the binding of HDAC with 14-3-3 proteins for the cell death occurred by the combined treatment, we tested for cell death using HDAC4(3SA) mutant which doesn’t bind to 14-3-3 proteins. Surprisingly, the cell death still occurred with the combined treatment of VPA and TRAIL even with no binding of HDAC4 mutant with 14-3-3 proteins. Therefore, we came to a conclusion that the binding of HDAC4 and 14-3-3 has nothing to do with the cell death occurred by the combined treatment. (data not shown).

Our findings demonstrate that HDAC4 degradation plays an important role in cell death induced by the combined treatment of TRAIL and VPA in TRAIL-resistant cells. Therefore, we conclude that this drug combination may be an effective anticancer therapeutic agent to control head and neck cancer.

## Material and Methods

### Cell culture and reagents

FaDu was purchased from American Type Culture Collection (Manassas, VA), and SNU899 was obtained from Korean Cell Line Bank (Seoul, Korea). HN3 was a gift from Dr. Sang-Yoon Kim at Asan Medical Center, University of Ulsan College of Medicine. FaDu and HN3 were cultured in minimum essential medium (Gibco/Invitrogen, Carlsbad, CA) containing 1% nonessential amino acid and 1% sodium pyruvate, and SNU899 in RPMI1640 (Gibco/Invitrogen, Carlsbad, CA). Culture media were supplemented with 10% fetal bovine serum (heat-inactived), and antibiotics (1%, Gibco/Invitrogen, Carlsbad, CA). Cell cultures were incubated at 37 °C in a humidified atmosphere of 5% CO_2_. Valproic acid (VPA) was purchased from Calbiochem (San Diego, CA, USA). TRAIL was obtained from professor Yong-Sung Kim (Ajou University, Korea). The pan-caspase inhibitor (Z-VAD-FMK), caspase-3 inhibitor (Z-DEVD-FMK), caspase-6 inhibitor (Ac-VEID-FMK) and caspase-9 inhibitor (Z-LEHD-FMK) were purchased from Santa Cruz Biotechnology (Santa Cruz, CA).

### MTT assay

Cells were seeded at a density of 3 × 10^4^ cells/ml in 96-well plates. 24 h post-seeding, cells were treated with TRAIL and/or VPA for 24, 48 and 72 h. Subsequently, MTT (3-(4,5-dimethylthiazol-2-yl)-2,5-diphenyl-tetrazolium bromide) (Sigma, St. Louis, MO) solution was added, and the data are presented as a percentage relative to control cells. (*p < 0.05; **p < 0.01; ***p < 0.001).

### Annexin-V and Propidium iodide (PI) staining assay

Annexin-V/PI staining assay (BD Biosciences, San Jose, CA) was performed as previously described^32^. HN3 cells were plated at 1 × 10^6^ cells/well in 6-well plates and were treated with TRAIL and/or VPA. After 24 h of cell seeding, the apoptotic cells were stained with Annexin-V-FITC and PI solution, and the stained cells were analyzed via flow cytometry (BD Biosciences).

### Western blotting

Cells were lysed with RIPA buffer (25 mM Tris-HCl pH 7.6, 150 mM NaCl, 1% NP-40, 1% sodium deoxycholate, 0.1% SDS) (Sigma) containing protease and phosphatase inhibitor cocktails (Roche). Western blotting was performed using the following antibodies; DR4, DR5 (Koma biotech, South Korea), and Bax, XIAP, Bid, PARP, cleaved caspase 8, 7, 3, HDAC1, 2, 3, 4, 5, 7, 14-3-3 proteins (alpha/beta, gamma, epsilon, eta, tau and zeta) and a-tubulin (Cell signaling technology, CA). α-tubulin, β-actin, or GAPDH were used as loading controls.

### Cell surface receptor expression

Cells were washed with PBS and incubated with anti-DR4 or DR5 antibody (R&D Systems, Minneapolis, MN) in PBS supplemented with 2% BSA, for 30 min at 4 °C. Cells were washed twice further with PBS and incubated with secondary anti-goat antibody conjugated with FITC (Pierce, Rockford, IL) for 30 min at 4 °C. Cells were then analyzed by flow cytometry (BD Biosciences).

### siRNA transfection

Transfection with siRNA was conducted as previously described^17^. The HN3 cells were transfected with siRNA (100 nM) using RNAiMAX (Invitrogen, CA). After 24 h of siRNA transfection, the cells were treated with TRAIL and/or VPA.

### Statistical analysis

Statistical evaluation of the data was conducted by Student’s *t* test. The results were considered as statistically significant with p < 0.05.
